# More than visual literacy: art and the enhancement of tolerance for ambiguity and empathy

**DOI:** 10.1186/s12909-017-1028-7

**Published:** 2017-11-10

**Authors:** Miriam Ethel Bentwich, Peter Gilbey

**Affiliations:** Faculty of Medicine, Bar-Ilan University, Safed Campus, P.O. Box 1589, Ramat Gan, Israel

## Abstract

**Background:**

Comfort with ambiguity, mostly associated with the acceptance of multiple meanings, is a core characteristic of successful clinicians. Yet past studies indicate that medical students and junior physicians feel uncomfortable with ambiguity. Visual Thinking Strategies (VTS) is a pedagogic approach involving discussions of art works and deciphering the different possible meanings entailed in them. However, the contribution of art to the possible enhancement of the tolerance for ambiguity among medical students has not yet been adequately investigated. We aimed to offer a novel perspective on the effect of art, as it is experienced through VTS, on medical students’ tolerance of ambiguity and its possible relation to empathy.

**Methods:**

Quantitative method utilizing a short survey administered after an interactive VTS session conducted within mandatory medical humanities course for first-year medical students. The intervention consisted of a 90-min session in the form of a combined lecture and interactive discussions about art images. The VTS session and survey were filled by 67 students in two consecutive rounds of first-year students.

**Results.:**

67% of the respondents thought that the intervention contributed to their acceptance of multiple possible meanings, 52% thought their visual observation ability was enhanced and 34% thought that their ability to feel the sufferings of other was being enhanced. Statistically significant moderate-to-high correlations were found between the contribution to ambiguity tolerance and contribution to empathy (0.528–0.744; *p ≤ 0.01*).

**Conclusions:**

Art may contribute especially to the development of medical students’ tolerance of ambiguity, also related to the enhancement of empathy. The potential contribution of visual art works used in VTS to the enhancement of tolerance for ambiguity and empathy is explained based on relevant literature regarding the embeddedness of ambiguity within art works, coupled with reference to John Dewey’s theory of learning. Given the situational nature of the tolerance for ambiguity in this context, VTS provides a path for enhancing ambiguity tolerance that is less conditioned by character traits. Moreover, the modest form of VTS we utilized, not requesting a significant alteration in the pre-clinical curricula, suggests that enhancing the tolerance of ambiguity and empathy among medical students may be particularly feasible.

## Background

Visual Thinking Strategies (VTS) is a pedagogic approach involving discussions of works of art aimed to encourage learners to look carefully, verbalize their observations and ideas, and interact with others regarding their interpretations of the images [1]. A key common goal for employing VTS classes is the enhancement of visual observation or visual literacy [1–5], which is the ability to ‘read,’ interpret, and understand information presented in pictorial or graphic images [6].

VTS, as a pedagogic approach concerning the discussion of art works in medical education, can also be placed within the larger context of medical humanities studies in medical schools. These studies pertain to realms such as literature, narrative, poetry, theater, and visual arts in medical educational programs [7, 8]. Incorporating various medical humanities courses into the curriculum of medical schools is aimed to offer students practical tools for self-reflection and communication with patients, along with an increased sense of empathy [9–11]. Accordingly, works of art teaching, such as the case of VTS, is understood as potentially contributing not merely to visual literacy and visual diagnostic skills of students but also to their ability for self-reflection, communication skills with patients (and colleagues) and an increased sense of empathy [1, 3, 12, 13].

Furthermore, VTS specifically revolves around students’ exposure to different interpretations of the same art image. Such focus corresponds with the definition for tolerance of ambiguity as the recognition of the option to interpret something in two or more distinct ways [14]. In a similar vein, a pivotal Geller and colleagues constructed a pivotal 4-item scale designed to measure the tolerance (or lack of it) to ambiguity among medical students [15]. According to this scale, respondents who prefer a job, problem or medical sub-profession *allowing multiple interpretations* are considered to be more tolerant of ambiguity.

At the same time, much importance is attributed to the tolerance of ambiguity in the context of professional medical care. Tolerating ambiguities and knowing how to manage them is said to be one of the conditions for being a medical expert [16, 17]. Ambiguities are often inherent in the information provided to the physician. Professional competence necessitates managing situations characterized by incomplete information, where there is no single clear answer or a correct course of action [18, 19]. Thus, the daily routine of doctors is characterized by many situations in which there is no such single clear answer or a correct course of action. Whether it is because there are multiple options for diagnosis, or because it is hard to anticipate patients’ reactions to treatments, or given the interpersonal differences among physicians with regard to their attitudes, values and perceptions of risk [20, 21]. Yet, medical students tend to think of medical knowledge as either absolutely certain, namely as offering clear-cut answers, or only temporarily uncertain, until a clear-cut answer will be found [22–25]. Other studies found that working under conditions of continuous ambiguity, in which there is lack of one clear answer, causes stress especially among junior physicians, as opposed to more experienced physicians [26–28].

In a similar vein, empathy is considered a crucial factor in the establishment of a good patient-clinician bond [29]. Empathy is the ability to truly understand another individual’s personal experiences and feelings, as well as his view of the world around him; hence the ability to empathize is based on both cognitive and emotional competencies [30]. In the context of healthcare provision, the medical expert’s empathy is reflected in his efforts to understand the patients’ subjective experiences without the need to join them [31, 32]. The importance attributed to empathy in the context of the medical domain may explain why previous studies concerning VTS and other visual fine arts interventions focused on demonstrating the possible enhancement of medical students’ empathic ability.

Moreover, it has been found that in the counseling context, empathic understanding of a patient is significantly correlated to the counselor’s tolerance of ambiguity [33]. In fact, another study showed that a physician’s ability to acknowledge patients’ emotional distress is an essential first step in initiating a discussion of medically challenging issues with no unequivocal solution [34]. Indeed, it is said that, in ambiguous situations, clinicians not only have to empathize with their patients’ concerns, but also express empathy in the face of the uncertainty they experience themselves [35].

However thus far, there has been scarce reference in the literature about the connection of visual arts classes (like VTS) to an increased tolerance of ambiguity and its possible correlation to empathy. One recent study that did attempt to examine the contribution of VTS to enhancing the students’ tolerance for ambiguity did not produce evidence for a substantial contribution in this realm [36, 37]. In another study, students were asked to evaluate the extent to which a visual art program had achieved its goal. While one of the goals with which the students were presented was related to tolerance for ambiguity, the students’ scores were given aggregately to all the goals together, rather than specifically to tolerance for ambiguity [38].

Furthermore, tolerance of ambiguity is still often characterized as either a personality trait or a phenomenon related to personality traits [39–42]. In fact, within the context of medical education, it was recently suggested that medical schools frequently ignore the importance of ambiguity tolerance and that therefore they should incorporate ambiguity tolerance measurement(s) as part of the selection process of their candidates [43]. However, an alternative approach to this issue may be offered by suggesting that the discussion of art works, through the application of VTS, may contribute to medical students’ tolerance of ambiguity. Such approach, will allow acknowledging the significance of ambiguity tolerance among future physicians, while suggesting a path for the development of this tolerance instead of dictating a less egalitarian admission process grounded on personality traits. Indeed this approach seems to be supported by research findings that depicts tolerance to ambiguity as a state rather than a trait, thereby emphasizing its context-dependent or situational nature [23, 44]. Finally, there is a wide range of length of VTS activities offered to medical students, including merely a single session format [4, 45–47]. Consequently, VTS as a form of visual arts teaching specifically offers a potential educational vehicle for the enhancement of students’ tolerance of ambiguity, without requiring substantial financial investment or significantly altering the overall curricula in medical school.

The present study offers a novel perspective on the contribution of visual arts teaching (through VTS) to medical students’ tolerance for ambiguity, in the sense of *accepting multiple interpretations*. The study also examines the correlation between this contribution of visual arts teaching and its contribution to empathy. Integrating the results gained in the study together with relevant theoretical literature, the article offers theoretical explanations for these findings, thereby highlighting the two-fold potential importance of visual arts teaching via the VTS approach for the enhancement of ambiguity tolerance. Hence, the importance of such teaching contribution to the tolerance of ambiguity is stressed for itself and due to its potential contribution to empathy. Moreover, we purposely chose to employ a relatively modest form of VTS intervention, neither requiring significant alterations in the pre-clinical curricula, nor necessitating substantial financial or educational efforts. This way, the potential contribution of visual arts teaching to the toleration of ambiguity and empathy will be examined under settings that could be easily employed in (almost) any medical school.

## Methods

### Participants and sampling

The VTS intervention examined in the current study was conducted within the mandatory medical humanities and medical ethics course for first-year medical students, in which 60 students are enrolled each year. The intervention was conducted during two consecutive academic years, including 120 students. 67 students filled out the short survey following the intervention (response rate = 56%). In the first year we had a relatively lower rate of participation (*n* = 17; 28%), and therefore decided to repeat the intervention in the following year (*n* = 50; 83%).

It should be noted that the extremely lower rate of participation in the first cohort might be misleading. Hence, it seems to be related to particular winterish weather conditions, resulting in the absence of many students on that day, so that practically only 35 students (out of the 60 students enrolled in the course) were present in the intervention. In other words, the lower rate of participation does not necessarily reflect an extreme selection bias, in which mainly the students who were more interested in the subject of the intervention completed the survey. It should also be noted that some previous pivotal published studies concerning VTS interventions also had a low response rate of around 30% [5, 38], resembling our first year’s response rate. In fact, in another recently published study this rate was as low as 2% [36, 37]. Furthermore, other VTS and visual arts based programs were conducted within the framework of either selective or elective courses. In contrast, our intervention was performed within a mandatory course, in which all first-year students are enrolled. Consequently, the student population, 56% of whom completed the survey, was potentially less homogenous, insofar as their attitude towards art within the medical education curricula is concerned, in comparison to students enrolled in selective or elective courses.

### Procedure and instrument

We conducted a two-academic-hours (90 min) intervention in the form of a combined lecture and interactive discussion about art images. The intervention was repeated during two consecutive years in the same format, and was led by one of the paper’s authors, who is a senior physician with an interest in art. Primary recommendation regarding questions that should be asked during VTS sessions was followed [48]. Additional information regarding the intervention is found in Table 1. At the end of the class, students were asked to fill out a short anonymous survey regarding the VTS intervention (see VTS Survey Supplementary file).

**Table 1:** Main characteristics of the VTS intervention

• The VTS intervention consisted of a single 90-min class within the Medical Humanities mandatory course. This class repeated separately in two different but consecutive cohorts of first-year students within our graduate program for medical students.
• In both years, the class took place roughly in the beginning of the second semester, after students had already completed their Gross Anatomy course, as well as the Public Health course and a basic 3-day module of introduction to medical humanities.
• The Medical Humanities mandatory course runs throughout the two pre-clinical years, consisting of classes in medical ethics, psychology, history of medicine sociology and art (in addition to the VTS intervention).
• The VTS intervention included 5 images, mainly images from the modern era, depicting situations in which sick, dying or deceased patients were involved (e.g. “The Anatomy Lesson of Dr. Nicolaes Tulp by Rembrandt [1632]”, “The Doctor” by Sir Luke Fildes, [1891], “Death in the Sickroom” by Edvard Munch [1894], etc.)
• Before displaying the images, the lecturer gave a brief explanation regarding the idea of VTS and its application in other medical schools (approx. 15 min).
• Each image was displayed on a big screen. All images were in color.
• Following the primary recommendation regarding the questions that should be asked during VTS sessions, we followed the three-question scheme suggested in the literature regarding VTS (see reference no. 33), about which we elaborate below.
• Students were initially presented with the first question: *what was going on in the displayed image*.
• Students were then given 5 min to look, examine and reflect about each image with their friends.
• Afterwards, the students were asked to share aloud their thoughts about what was going on in the art image with the lecturer and their classmates.
• For each depiction offered by a student regarding the displayed image, the lecturer asked a second question, namely, “*what do you see [in the image] that make you think that?*” Other students were encouraged to join in the discussion as well.
• Finally, the lecturer also encouraged the students to examine *whether they could spot further interesting details in the picture*, thereby following the third recommended question for VTS sessions.
• The open discussion regarding each image lasted approximately 15 min.

The basic survey, repeated in both years, was comprised of four closed-ended questions (CQs) and one open-ended question. The CQs were comprised of statements about which the students were asked to rate their extent of agreement on a 5-point Likert Scale (5 = a great deal; 1 = not at all) regarding different aspects of the possible contribution of the intervention class to their training. These aspects of possible contribution are displayed in Table 2 below. In the open question, students were asked to write any comments they had about the VTS class, but given the low response rate to this question, it was omitted from our analysis.

**Table 2:** Aggregated Two Years’ Frequencies, percentages and cumulative percentages for four basic abilities^a^

Ability	N	%	Cumulative %
Accept multiple possible meanings (AMM)			
A great deal	7	10.5	10.4
Quite a bit	22	32.8	43.3
Some	16	23.9	**67.2**
A little	13	19.4	86.6
Not at all	9	13.4	100.0
Total	67	100.0	
Visually Observe (VB)			
A great deal	3	4.5	4.5
Quite a bit	15	22.4	26.9
Some	17	25.4	**52.2**
A little	21	31.3	83.6
Not at all	11	16.4	100.0
Total	67	100.0	
Feel the suffering of others (FSO)			
A great deal	1	1.5	1.5
Quite a bit	5	7.5	9.0
Some	17	25.4	**34.3**
A little	20	29.9	64.2
Not at all	24	35.8	100.0
Total	67	100.0	
Teamwork (TA)			
A great deal	1	1.5	1.5
Quite a bit	1	1.5	3.0
Some	9	13.4	**16.4**
A little	20	29.9	46.3
Not at all	36	53.7	100.0
Total	67	100.0	

In addition, in the second year we included two more CQs relating to empathy from two additional perspectives (See Table 3 below). As the “Results” section shows, students in the first cohort attributed to the VTS session relatively lower contribution to their empathy, as opposed to previous studies. Therefore, we wished to explore whether additional aspects of empathy might change the results. Specifically, we speculated that, given VTS’ emphasis on “observing” images, framing empathy in terms of “observing other people’s feelings” may be more connected to the activity entailed in VTS.

**Table 3:** Frequencies and percentages for different aspects of empathy (2016)

Ability	N	%	Cumulative %
Observe the feelings of others			
A great deal	0	0	0
Quite a bit	7	0.14	0.14
Some	14	0.28	**0.42**
A little	12	0.24	0.66
Not at all	17	0.34	100.0
*Total*	*50*	*100.0*	
Understand the feelings of patients and their families			
A great deal	0	0	0
Quite a bit	5	0.10	0.10
Some	13	0.26	**0.36**
A little	13	0.26	0.62
Not at all	19	0.38	100.0
*Total*	*50*	*0.100*	
Feel the suffering of others			
A great deal	1	0.2	0.2
Quite a bit	3	0.6	0.8
Some	11	0.22	**0.30**
A little	14	0.28	0.58
Not at all	21	42.0	100.0
*Total*	*50*	*100.0*	

We used the *SPSS 21* software package in order to perform the statistical analysis, which included frequencies, percentages and correlation analyses. For the latter, Spearman Rho was employed, since all variables were on an ordinal scale.

## Results

In the current section, we present the main findings of the survey conducted after each VTS intervention class in 2015 and 2016, focusing on the closed-ended questions (CQs) alone. We begin by introducing the overall combined findings for both years, and then, given the gaps in the findings between the two years, we focus on each year separately, including the additional 2 questions added regarding empathy on the second year.

Table 2 shows the overall combined frequencies (number of participants and percentages) of support that students expressed regarding the four main examined domains in both years together. In what follows we summarize the cumulative results concerning the contribution students attributed to discussions of art works during the VTS intervention. We omit from this summary the percentages of students indicating that the VTS intervention had only contributed “a little” or “not at all,” since we understand these two lower scores to be within the negative range of the scale (1–2). It is also important to stress at this point that our students are familiar with this scale from their regular course feedback scheme used in our medical school for all courses, in which the students are accustomed to indicate the lower scores (1–2) as a negative feedback.

As many as 67% of the students (*n* = 45) thought that the discussion of art works contributed to their acceptance of multiple possible meanings, thereby implicitly recognizing the existence of ambiguities. Similarly, though to a lesser degree, 52% (*n* = 35) thought that this discussion contributed to their visual observation ability. However, with respect to notions relating to empathy (“feel the sufferings of others”) and the student’s teamwork ability, the number and percentages of students supporting these notions were substantially lower. Only 34% of the students (*n* = 23) thought that the VTS class contributed to their ability to feel the suffering of others; and only 16% of the students (*n* = 11) expressed support for the idea that the class contributed to their teamwork ability. It should be noted, though, that the extremely lower support for the contribution of VTS to the teamwork ability (16%) seems to reflect the fact that our VTS class had put less emphasis on working in small groups. Hence, the time allocated for discussions in small groups was utterly limited (5 min for each image, as noted in Table 1 above). In addition, the general setup of the class was oriented towards large-classroom setting, so that the small-groups sessions were conducted between students seated on the same row and isle, within the same large open space in which the whole class was situated.

**Fig. 1:**
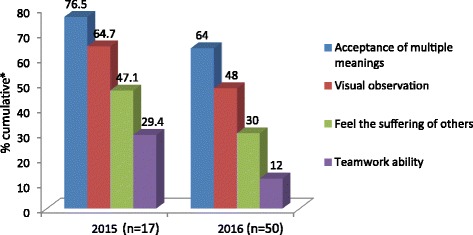
Cumulative percentages of perceived VTS contribution to main domains. * Cumulative refers to the percentage sums of the highest 3 scores for each variable (values 3–5)

Admittedly, the data also reveal that there are differences between the two years in which the study was conducted with respect to the expressed attitudes of students regarding the contribution of the discussion of art works during the VTS intervention. Thus, Fig. 1 shows that among the 2015 class, the percentages of support for the contribution of VTS to the tolerance of ambiguity, visual observation and empathy were 76.5%, 64.7% and 47%, respectively. In contrast, the percentages of support for the VTS class’s contribution to these domains in the following year (2016) were 64%, 48%, and 30%, respectively. Despite these gaps, in both years, the contribution of VTS to the tolerance of ambiguity (i.e. acceptance of multiple possible meanings) and visual observation are reported by the students to be substantially higher than the contribution to empathy. In fact, as Table 3 shows, even when the additional aspects of empathy were introduced for the second cohort, the highest cumulative percentage of support for the contribution of VTS to empathy was still lower than its contribution to other domains. Hence, while 42% of the students supported the idea that VTS contributed to their ability to observe the feelings of others, this is still lower than the 64% and 49% of support offered regarding the contribution of VTS to ambiguity toleration and visual observation, respectively.

Finally, Table 4 presents the correlations between the four variables denoting the four basic questions presented in both years. The highest correlation was found between the contribution to observation ability and the contribution to acceptance of multiple possible meanings (.874, *p < =0.01*; .707, *p < =0.01* in 2015 and 2016, respectively). It is of interest to note that the correlation between the acceptance of multiple possible meanings and the understanding of the sufferings of others, as well as between the latter and visual observation ability are lower among the students of the 2015 class in comparison to the class of 2016. Hence, in 2015 the values of these correlations are .528 and .523, respectively (*p < =0.05* for both), whereas for the class of 2016, the correlations found for these variables are .661 and .645, respectively (*p < =0.01* for both). When the two additional aspects of empathy are introduced in the second year, the correlations are even higher, particularly with respect to “ability to observe the feeling of others” (.744 and .697; *p < =0.01* for both new aspects).

**Table 4:** Correlations between main variables

Year		AMM	VB	FSO	FO	UFP	TA
2015	Acceptance of multiple meanings (AMM)	1.000	.**874** ^**^	**.528** ^*^			.632^**^
	Visual observation (VB)	**.874** ^**^	1.000	.523^*^			.684^**^
	Feel the suffering of others (FSO)	**.528** ^*^	.523^*^	1.000			.621^**^
	Observe the feelings of others (FO)						
	Understand feelings of patients/families (UFP)						
	Teamwork ability (TA)	.632^**^	.684^**^	.621^**^			1.000
2016	Visual observation (VB)	**.706** ^**^	1.000	.645^**^	**.697** ^**^	.686^**^	.292^*^
	Acceptance of multiple meanings (AMM)	1.000	**.706** ^**^	.661^**^	**.744** ^**^	.630^**^	.290^*^
	Feel the suffering of others (FSO)	.661^**^	.645^**^	1.000	.869^**^	.848^**^	.461^**^
	Observe the feelings of others (FO)	**.744** ^**^	.**697** ^**^	.869^**^	1.000	.877^**^	.511^**^
	Understand feelings of patients/families (UFP)	.630^**^	.686^**^	.848^**^	.877^**^	1.000	.464^**^
	Teamwork ability (TA)	.290^*^	.292^*^	.461^**^	.511^**^	.464^**^	1.000

* p < =0.05 ** p < =0.01 2015: N = 17; 2016: *N* = 50. **Bold** font indicates values of interest

## Discussion

Tolerance of ambiguity, understood in this study as the “acceptance of multiple possible meanings” [14], was found to surpass all other main potential domains of contribution. This finding not only contrasts with previous studies, usually depicting visual literacy and visual observation as the most important domain of contribution that VTS offers [1, 4, 5, 13, 36, 37, 46], but is also important in itself. Hence, as noted previously, the nurturing of such ability among future physicians is of utmost importance, given the nature of the profession.

Our study also indicates that art works’ discussions as they are employed in VTS may have a potential contribution to empathy. However, it does so in a more complicated manner. This contribution was lower in comparison to the contribution of VTS to tolerance of ambiguity and visual literacy in both years; even the highest percentage of students supporting VTS contribution to one particular aspect of empathy in the second year (42%) still lags behind the contribution students attributed to VTS concerning ambiguity toleration and visual literacy. Yet, the results also depict a very high correlation between students’ attitudes regarding the contribution of VTS to their ability for observing the feelings of others and their attitudes regarding the contribution of VTS to tolerance of ambiguities. This strong correlation may serve as a further indication for the potential importance of visual art teaching entailed in VTS to the toleration of ambiguities because it is also related to the enhancement of empathic ability.

Nonetheless, in order to substantiate our hypothesis regarding the possible contribution of art classes (in the form of VTS) to the enhancement of medical students’ abilities to tolerate ambiguities and empathize, one further question should be explored. This is the question why art classes, such as the VTS intervention we employed in our research, may serve as an enhancer of medical students’ ability to tolerate ambiguities, and how such tolerance may be related to empathy.

Art of all sorts is said to be inherently ambiguous, because what the artist intended to express can never be known for sure by the audience [8–10, 49, 50]. Ambiguity typifies various art forms: in literature, in music and in the visual arts [51]. In fact, it has been recognized that ambiguity is associated with the participation or involvement of the audience in the artworks and evaluations of their aesthetic qualities [51]. Ambiguous artworks offer especially fine visual material to facilitate good thinking habits among students, and can become indelible visual experiences for students as they learn to think critically about these pieces [52].

Furthermore, we suggest that there is a good reason why tolerance of ambiguity, embedded within art, is related to an enhanced empathic ability. Building on John Dewey’s theory of learning, Andrea English has recently portrayed a manner by which the use of arts is directly linked to enhanced empathy and tolerance of ambiguity [53]. Thus, she stresses Dewey’s concept of “Imagination” and the way in which it is connected to art and empathy. The capacity for imagination has a key role in Dewey’s learning theory since this capacity facilitates the transformative learning process, happening through our interactions with others. Consequently, imagination elaborates and furthers our thinking, such that we are able to consider and absorb that which is beyond our own mind’s knowledge and perception. Yet by being able to consider and absorb things that are beyond our own knowledge and perception, the understanding that there may be multiple meanings and interpretation to the same situation settles in as well. Hence, in terms of tolerance for ambiguity, we may comprehend the imagination as a vehicle for the enhancement of this tolerance. Cultivating the imagination, in turn, necessitates rich experiences embedded in learning environments, which will broaden our horizons and extend our thinking. In fact, Dewey contends that the arts enable us to “enter, through imagination and the emotions they evoke, into other forms of relationships and participation than our own” ([54] p. 336). It follows that since the arts are a potential significant cultivator of imagination, they may be understood as vital for the enhancement of the tolerance for ambiguity.

Moreover, it is precisely through our understanding that the arts (and imagination) are means to enhance the tolerance of ambiguity, that we may also learn to appreciate their importance for the enhancement of empathy. According to this interpretation, empathy necessitates the ability to see ‘the world’ from the other person’s viewpoint, while keeping well-drawn boundaries between self and other. Yet such ability is contingent upon acknowledging that there can be other views of the world or multiple interpretations of it, namely that we are able to tolerate ambiguities [53]. Therefore, from this perspective, empathy and tolerance of ambiguity are closely related.

This close linkage seems to be also echoed from a grammatical standpoint. The concept of empathy, according to Helen Reis was initially introduced in the mid-nineteenth century by aestheticians, using the German word “Einfühlung” to “describe the emotional ‘knowing’ of a work of art from within, by feeling an emotional resonance with the work of art” ([55] p. 75). Hence, it seems that the term “empathy” originated from the idea that observing closely works of art allows the viewer to feel an emotional resonance with these art works.

In this sense, the very strong correlation we found between students’ attitude towards the contribution of VTS to empathy and the contribution of VTS to the tolerance of ambiguity resonates empirically with the above theoretical explanation regarding the linkage between tolerance for ambiguity and empathy. This being the case, our study’s results, reinforce the importance of discussing works of art not merely for the tolerance of ambiguity, but also for the potential importance of this contribution to the enhancement of students’ empathy as well.

Finally, through the suggested linkage between the contribution of VTS to ambiguity toleration and its contribution to empathy, our study may also have implications regarding the nature of ambiguity tolerance in the context of these classes. Hence, if the ability to empathize with the patients and/or families portrayed in the art images allows the students to better understand the ambiguities entailed in the depicted *situations* conveyed in the art images, it seems that students are engaged in *situational* tolerance of ambiguity. This latter form of ambiguity toleration, which as noted in the “Introduction” section, is also recognized in the theoretical literature regarding this subject stands in contrast to the depiction of tolerance for ambiguity as a personality trait or related to it [25, 40, 56]. In other words, VTS as a pedagogic approach for discussing works of art may offer an alternative path for enhancing the tolerance of ambiguity. Such path need not necessarily be contingent upon predetermined students’ personality traits, thereby further stressing the special contribution this approach for visual arts teaching may have in the context of enhancing the tolerance for ambiguity among medical students.

### Study limitations

Similarly to other studies in this realm [12, 38, 45], our findings are based on students’ self-reports and subjective perceptions regarding the visual experience. Hence their evaluations should be treated with caution. That being said, we ran the study during two consecutive academic years with two different groups of students and still got similar rankings for the contribution of VTS to the different examined domains. That is, as Fig. 1 showed, in both years, VTS had the largest contribution to the toleration of ambiguity, followed by its contribution to visual observation, empathy, and having the least contribution to teamwork. Therefore, the foundations of these results seem to be relatively strong.

Another limitation of the research is that it lacks a control group. While we certainly recognize this limitation, given the fact that the research was set in a mandatory course, depriving some of the students of the content of the VTS class seemed unethical to us. Furthermore, some of the previous published studies of the potential contribution of VTS to medical students also refrained from having a control group precisely for this reason [1, 4, 38].

Finally, we acknowledge the fact that the scale employed in our study has the limitation of not providing the respondent with the opportunity to indicate that the VTS intervention has decreased or worsened their various self-reported abilities. Hence, students were confined to responses revolving around the extent of contribution that VTS offers to the examined abilities, without an option to report on the possible damage(es) that this intervention had caused. However, we suspect that in the setting of a self-reported survey done for a one-time (relatively) short intervention, employed in the study, the students’ ability to evaluate possible damages is quite limited in the first place. It might be the case, though, that in future research pertaining to longer interventions allowing an objective pre/post testing design, instead of self-reported surveys, such an evaluation would be more applicable.

## Conclusions

The possibility that visual art images may contribute to the tolerance of ambiguity has thus far either received less attention or alternatively has been depicted as unsupported. Our study has shown that visual art used in VTS may contribute to developing medical students’ tolerance of ambiguity, and that the latter is also related to empathy enhancement. The potential contribution of visual art works used in VTS to the enhancement of tolerance for ambiguity and empathy is explained based on relevant literature regarding the embeddedness of ambiguity within art works, coupled with reference to John Dewey’s theory of learning. Due to the situational nature of the tolerance for ambiguity in this context, it may also provide a path for enhancing ambiguity tolerance that is less conditioned by character traits. Also, given the modest form of VTS we utilized, enhancing the tolerance of ambiguity and empathy among medical students seems particularly feasible. Future research regarding VTS and other approaches for exposing students to works of art in medical schools, which employ an objective pre/post design, is needed in order to corroborate (or refute) our findings.

## Abbreviations

CQs: closed-ended Questions
VTS: Visual Thinking Strategies
