# Correction to: More than visual literacy: art and the enhancement of tolerance for ambiguity and empathy

**DOI:** 10.1186/s12909-017-1088-8

**Published:** 2017-12-21

**Authors:** Miriam Ethel Bentwich, Peter Gilbey

**Affiliations:** Faculty of Medicine, Bar-Ilan University, Safed Campus, P.O. Box 1589, Ramat Gan, Israel

## Correction

Following publication of the original article [1], the authors reported that the corrections they had requested for Table 3 had not been implemented, and that the title for Table 2 included an unnecessary indication for remark/reference (“a” in a superscript font) at the end of the title. Also, the affiliation of the authors had not been clearly stated: it should read ‘Faculty of Medicine, Bar-Ilan University, Safed Campus, P.O.Box 1589, Safed, Israel’.

The tables should appear as follows:

**Table 2:** Aggregated Two Years’ Frequencies, percentages and cumulative percentages for four basic abilities

Ability	*N*	%	Cumulative %
Accept multiple possible meanings (AMM)			
A great deal	7	10.5	10.4
Quite a bit	22	32.8	43.3
Some	16	23.9	**67.2**
A little	13	19.4	86.6
Not at all	9	13.4	100.0
*Total*	*67*	*100.0*	
Visually Observe (VB)			
A great deal	3	4.5	4.5
Quite a bit	15	22.4	26.9
Some	17	25.4	**52.2**
A little	21	31.3	83.6
Not at all	11	16.4	100.0
*Total*	*67*	*100.0*	
Feel the suffering of others (FSO)			
A great deal	1	1.5	1.5
Quite a bit	5	7.5	9.0
Some	17	25.4	**34.3**
A little	20	29.9	64.2
Not at all	24	35.8	100.0
*Total*	*67*	*100.0*	
Teamwork (TA)			
A great deal	1	1.5	1.5
Quite a bit	1	1.5	3.0
Some	9	13.4	**16.4**
A little	20	29.9	46.3
Not at all	36	53.7	100.0
*Total*	*67*	*100.0*	

**Table 3:** Frequencies and percentages for different aspects of empathy (2016)

Ability	*N*	%	Cumulative %
Observe the feelings of others			
A great deal	0	0	0
Quite a bit	7	14.0	14.0
Some	14	28.0	**42.0**
A little	12	24.0	66.0
Not at all	17	34.0	100.0
*Total*	*50*	*100.0*	
Understand the feelings of patients and their families			
A great deal	0	0	0
Quite a bit	5	10.0	10.0
Some	13	26.0	**36.0**
A little	13	26.0	62.0
Not at all	19	38.0	100.0
*Total*	*50*	*100.0*	
Feel the suffering of others			
A great deal	1	2.0	2.0
Quite a bit	3	6.0	8.0
Some	11	22.0	**30.0**
A little	14	28.0	58.0
Not at all	21	42.0	100.0
*Total*	*50*	*100.0*	

The original article has been corrected.
